# Regulation of Tripartite Motif-Containing Proteins on Immune Response and Viral Evasion

**DOI:** 10.3389/fmicb.2021.794882

**Published:** 2021-12-01

**Authors:** Xiu-Zhong Zhang, Fu-Huang Li, Xiao-Jia Wang

**Affiliations:** ^1^Key Laboratory of Animal Epidemiology of the Ministry of Agriculture, College of Veterinary Medicine, China Agricultural University, Beijing, China; ^2^Beijing General Station of Animal Husbandry Service (South Section), Beijing, China

**Keywords:** TRIM proteins, type I interferon, NF-kappa B, immune evasion, signaling pathways, immune response

## Abstract

Tripartite motif-containing proteins (TRIMs), exhibiting ubiquitin E3 ligase activity, are involved in regulation of not only autophagy and apoptosis but also pyrotosis and antiviral immune responses of host cells. TRIMs play important roles in modulating signaling pathways of antiviral immune responses *via* type I interferon, NF-κB, Janus kinase/signal transducer and activator of transcription (JAK/STAT), and Nrf2. However, viruses are able to antagonize TRIM activity or evenly utilize TRIMs for viral replication. This communication presents the current understanding of TRIMs exploited by viruses to evade host immune response.

## Introduction

**Figure 1:**
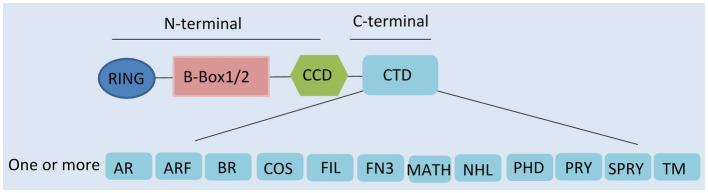
Domain structure of tripartite motif-containing protein (TRIM) family proteins. TRIM contains a RING domain, a B-box 1 and/or B-box 2 domain, a coiled-coil domain (CCD), and distinct C-terminal domains. AR, acid-rich region; ARF, ADP ribosylation-like factor; BR, bromodomain; COS, C-terminal subgroup one signature; FIL, filamin-type immunoglobulin; FN3, fibronectin type 3; MATH, meprin and tumor-necrosis receptor-associated factor homology; NHL, NHL repeats; PHD, plant homeodomain; PRY, SPRY-associated domain; SPRY, SPIa, and the ryanodine receptor domain; and TM, transmembrane.

Tripartite motif-containing proteins (TRIMs), an expanding family of proteins characterized by their N-terminal domains, containing a tripartite motif, are widely present in mammals (Marin, 2012). They are also known as RBCC proteins from the presence of an RBCC motif, consisting of a RING domain, one or two B-boxes, and a coiled-coil region (Figure 1; Reddy and Etkin, 1991; Meroni and Diez-Roux, 2005). In contrast to the conserved N-terminal domains, the additional C-terminal domains are variable and can be used to classify TRIMs into 11 subfamilies (Ozato et al., 2008).

Presenting in most TRIMs, the RING domain is comprised of a zinc finger motif (Borden, 2000). This motif confers E3-ligase activity and is able to catalyze the conjugation of ubiquitin and ubiquitin-like proteins (ISG15 or SUMO), leading to the degradation of targeted proteins (Meroni and Diez-Roux, 2005; Ozato et al., 2008; Marin, 2012). Following the RING domain, the B-box domains are also zinc finger motifs. However, their functions are still largely unclear (Nisole et al., 2005; Meroni, 2012). It seems that they are involved in viral recognition, self-association, or interactions with other proteins (Nisole et al., 2005; Meroni, 2012). The coiled-coil domain (CCD) is the third domain of the RBCC motif. This domain has the ability to assemble with other coiled-coil structures, mediating homomeric self-association and heteromeric assemblies (Nisole et al., 2005). So far, 10 different types of C-terminal domain have been described, one or more of which can be present (Ozato et al., 2008). Specific C-terminal domains confer different functions by recruiting unique functional partners (Nisole et al., 2005; Khan et al., 2019).

TRIMs have been characterized in detail as members of E3-ubiquitin ligase group according to their interactions with E2-ubiquitin ligase (Rajsbaum et al., 2014a). Briefly, TRIMs transfer ubiquitin to the target protein by facilitating interaction with E2 enzymes via their RING domain. It has been shown that TRIMs can catalyze synthesis of K48-, K63-, or unanchored K63-linked poly-ubiquitin chains. Proteins that with K48-linked poly-ubiquitin are usually targeted for degradation by the proteasome. However, proteins with K63-linked polyubiquitin are involved in activation of antiviral signaling pathways. In addition, unanchored K63-linked poly-ubiquitin chains have also been proposed to activate kinases involved in signaling pathways in a proteasomal degradation-independent manner.

Tripartite motif-containing proteins are involved in various biological functions, including apoptosis, pyroptosis, antiviral activity, and viral evasion (Hatakeyama, 2017; Chen et al., 2018b; Koepke et al., 2021). TRIMs are involved in innate immune responses to restrict the replication of various viruses, while viruses have evolved strategies to antagonize TRIMs (Nisole et al., 2005; Rajsbaum et al., 2014a; Koepke et al., 2021). In addition, viruses are able to employ TRIMs to enhance their replication (Xing et al., 2017; Li et al., 2018; Zheng et al., 2019a).

In this review, we describe the functions of TRIMs in immune signaling pathways and discuss the latest research on viral evasion of immune system by antagonizing or utilizing TRIMs.

## Trim Regulation of Innate Immune Response

In response to viral infection, eukaryotes have evolved many strategies to restrict viral replication. As the first line of defense, the innate immune response is initiated when pattern recognition receptors (PRRs) recognize pathogen-associated molecular patterns (PAMPs; Medzhitov, 2007). There are a number of types of PRRs, including Toll-like receptors (TLRs), NOD-like receptors (NLRs), C-type lectin receptors (CLRs), and RIG-I-like receptors (RLRs; Kagan and Barton, 2014). PRRs use various adaptor proteins to activate downstream signaling pathways, and this leads to the production of interferons (IFNs) and inflammatory factors as well as interferon-stimulated genes (ISGs); those factors are important in the defense against incoming pathogens (Rajsbaum et al., 2014a).

Some review articles demonstrated that TRIMs act as important positive and negative modulators of PRR signaling pathways (Ozato et al., 2008; Rajsbaum et al., 2014a). In this section, we detail the role of TRIMs in regulating several main pathways, including TLR, RLR, STING, and Janus kinase/signal transducer and activator of transcription (JAK/STAT) signaling.

### Toll-Like Receptor

Human TLR1, TLR2, TLR4, TLR5, and TLR6 are associated with the plasma membrane, while TLR3, TLR7, TLR8, and TLR9 are located in the endosome (Jimenez-Dalmaroni et al., 2016; Odendall and Kagan, 2017). Although TLR4 is mainly located in the plasma membrane, it can also be internalized in the endosome (van Tol et al., 2017). TLRs were first identified as receptors of bacterial PAMPs and also have recently been found to respond to viral infection (Bottermann and James, 2018; van Gent et al., 2018). TLR3 senses both double-stranded RNA (dsRNA) and single-stranded RNA (ssRNA) of viruses, TLR7 and TLR8 can recognize viral single-stranded RNA, and TLR9 recognizes CpG motifs (Thompson et al., 2011; Tatematsu et al., 2013; Jimenez-Dalmaroni et al., 2016). All TLRs except TLR3 signal through the adaptor molecule MyD88 by recruiting the kinases IRAK1/4 and E3 ubiquitin ligase TRAF6, and this results in the activation of the NF-κB and AP-1 signaling pathways (Villano et al., 2014; Jimenez-Dalmaroni et al., 2016). TLR3 and TLR4 can interact with adaptor TRIF, resulting in the activation of IRF, NF-κB, and AP-1 signaling pathways *via* TRAF3 and TBK1/IKKε (Gay et al., 2014).

Tripartite motif-containing proteins are able to modulate the TLR signaling pathways (Shen et al., 2012; Hu et al., 2014; Sundquist and Pornillos, 2018; Ganser-Pornillos and Pornillos, 2019). It has been proposed that TRIM5α is implicated in NF-κB signaling pathways and promotes the synthesis of unanchored K63-linked polyubiquitin chains, which can in turn activate the TAK1 (Sundquist and Pornillos, 2018; Ganser-Pornillos and Pornillos, 2019). TRIM56 has been shown to interact with TRIF in TLR3-mediated IFN/ISG production (Shen et al., 2012). TRIM38 negatively regulates TLR signaling by targeting TRAF6, TRIF, and NAP1 for degradation, leading to the suppression of TAB2/3, TBK1/IKKε, and IRF3/7 (Zhao et al., 2012; Hu et al., 2014, 2015). TRIM8 negatively affects TLR3/4-mediated response by catalyzing the polyubiquitination of TRIF, resulting in disruption of the TRIF–TBK1 interaction (Ye et al., 2017).

### RIG-I-Like Receptor

RIG-I-like receptors are essential sensors in the process of viral infection, responding to dsRNA or ssRNA containing 5'-triphophates (Runge et al., 2014). RIG-I and melanoma differentiation-associated protein (MDA5) induce antiviral pathways and contain a central DEAD (Asp-Glu-Ala-Asp) box, a C-terminal domain (CTD), and two N-terminal caspase-activated recruitment domains (CARDs; Chan and Gack, 2015). RIG-I encoded by *Ddx58* binds small dsRNA, while MDA5 encoded by *Ifih1* recognizes long ssRNA (Chiang et al., 2018b; van Gent et al., 2018). Binding viral RNA to RIG-I or MDA5 induces the activation of RLR pathways. Then the CARDs are exposed, which leads to the recruitment of RIG-I or MDA5 to their common adaptor mitochondrial antiviral signaling protein (MAVS; Yoneyama et al., 2004; Jiang et al., 2012). MAVS can activate NF-κB and IRF3/7 pathways, finally *via* recruiting IKK-related kinases IKKα/β/γ complex and IKKε, respectively (Takeuchi and Akira, 2010).

Some TRIMs act as key regulators of RLR signaling (Tan et al., 2017; Chen et al., 2019; Sun et al., 2020). As a positive regulator of RLR signaling, TRIM35 directly catalyzes K63-linked polyubiquitination of TRAF3. This promotes the formation of a signaling complex TRAF3-MAVS-TBK1, facilitating the activation of IRF3/7 (Sun et al., 2020). TRIM14 provides a docking platform for the assembly of MAVS complex, consisting of Werner helicase interacting protein 1 (WHIP) and protein phosphatase PPP6C (Tan et al., 2017). TRIM14 is also reported to recruit adaptor protein IKKγ/NEMO to MAVS, taking part in RIG-1-mediated IRF3 and NF-κB signaling (Zhou et al., 2014). TRIM65, identified as an E3 ligase of MDA5, catalyzes K63-linked polyubiquitination of MDA5 at the RNA helicase domain K743, promoting IRF3/7 signaling to restrict encephalomyocarditis virus (EMCV; Lang et al., 2017). EcTRIM44L, identified from the orange spotted grouper fish, negatively regulates MAVS-mediated IFN response rather than IRF3-mediated (Zheng et al., 2019b).

### STING

STING is at the outer mitochondrial membrane and ER, and most cytosolic DNA sensors activate it. Various cytosolic DNA sensors have been identified in recent years, including cyclic GMP-AMP synthase (cGAS), DDX41, and DHDX36 (Ishikawa et al., 2009). It has been reported that they can detect various DNA viruses, including adenoviruses (AdV), human cytomegalovirus (HCMV), and HSV-1, as well as retroviruses such as vesicular stomatitis virus (VSV), human immunodeficiency virus (HIV), and Dengue virus (DENV; Paijo et al., 2016; Reinert et al., 2016; Sun et al., 2017; Tan et al., 2018). Upon DNA binding, STING signaling is activated. It has been shown that STING interacts with TBK1, which phosphorylates IRF3/7, resulting in type I IFN induction (Vance, 2016).

Several TRIMs have been demonstrated to modulate STING-mediated signaling *via* regulatory modifications (Tsuchida et al., 2010; Zhang et al., 2012; Wang et al., 2014; Seo et al., 2018). Mammalian TRIM32 may target STING for K63-linked ubiquitination to promote dimerization and enhance type I IFN production (Zhang et al., 2012). The function of TRIM 56 in the induction of antiviral response is controversial. An initial report described TRIM56 interacting with STING by inducing K63-linked polyubiquitination of STING upon cytosolic DNA stimulation, but a later study failed to detect any ubiquitination signal of STING in the presence of TRIM56. A very recent study showed that TRIM56 directly targets cGAS, rather than STING or its downstream signaling, against HSV-1 infection but not against influenza A virus (IAV) infection (Tsuchida et al., 2010; Wang et al., 2014; Seo et al., 2018), similar to the SUMOylation of RIG-I and MDA5, TRIM38 SUMOylates cGAS, and STING, which inhibits the ligation of K48-linked ubiquitin and proteasomal degradation (Hu et al., 2016).

### Janus Kinase/Signal Transducer and Activator of Transcription

Janus kinase/signal transducer and activator of transcription signaling induces multiple molecular immune responses and is essential in cytokine and growth factor signaling (Ghoreschi et al., 2009). Four different JAKs have been identified, including JAK1, JAK2, JAK3, and TyK2 (Tyrosine kinase 2), and seven members of STAT, including STAT1, STAT2, STAT3, STAT4, STAT5a, STAT5b, and STAT6 (Meyts and Casanova, 2021). JAK/STAT is initially activated by binding of cytokines, such as IFNα/β, IFNγ, and IL-6. IFNα/β employs TYK2 and JAK1 to phosphorylate STAT1 and STAT2, IFNγ recruits JAK1 and JAK2 to phosphorylate STAT1, and IL-6 leads to one or both JAK-mediated phosphorylations of STAT3 (Majoros et al., 2017; Banji et al., 2021). These activated STAT dimers are then transported to the nucleus and promote the transcription of interferon stimulated genes (ISGs; Raftery and Stevenson, 2017). Negative regulators, protein tyrosine phosphatase, non-receptor type 6 (PTPN6), suppressor of cytokine signaling (SOCS-1) and protein inhibitor of activated STAT (PIAS-1), suppress the JAK/STAT pathway (Cokic et al., 2012; Tikhe and Dimopoulos, 2021).

Reports have indicated that the JAK/STAT pathway can be regulated by some TRIMs (Rajsbaum et al., 2014b; Teng et al., 2020; van Tol et al., 2020). TRIM6 was found to modulate IFNα/β-induced JAK/STAT signaling for antiviral response *via* cooperation with E2-ubiquitin conjugase UbE2K and promotion of the synthesis of unanchored K48-linked polyubiquitin chains, which activated IKKε for subsequent STAT1 phosphorylation (Rajsbaum et al., 2014b). Another study identified VAMP8, a regulator of IFNα/β-induced JAK/STAT signaling, and mediator of the phosphorylation of STAT1 in West Nile virus infection. Its expression and function are dependent on TRIM6 activity (van Tol et al., 2020). TRIM59 interacts with STAT1 by recruiting much more PIAS1 to suppress the activation of STAT1, and it also suppresses IL-1β-induced activation of the JAK2/STAT3 pathway (Su et al., 2020; Teng et al., 2020). TRIM8 enhances IL-6-dependent activation of STAT3 by degradation of PIAS3, which is the protein inhibitor of activated STAT3 (Okumura et al., 2010).

## Viral Antagonization of Trims

**Figure 2:**
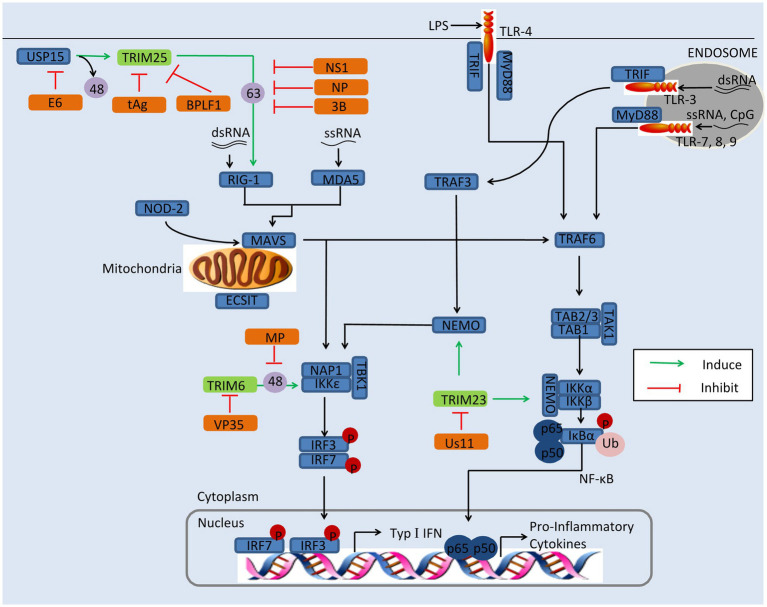
Viral evasion of immunity *via* antagonization of TRIMs. Some TRIMs (green) positively regulate innate immune response. Viral proteins (orange) antagonize these TRIMs to reduce the production of type I IFNs and inflammatory factors. BPLF1, the deconjugases of HSV; E6, the oncoprotein of HPV16; MP, the matrix structural protein of NiV; NS1, the nonstructural protein 1 of IAV; NSs, the nonstructural protein NSs of SFTSV; NP, the nucleocapsid protein of SARS-CoV, MERS-CoV, or SARS-CoV-2; tAg, the small t antigen of JCV; Us11, the Us11 protein of HSV-1; VP35, the VP35 protein of EBOV; 3B, the 3B protein of FMDV; 48, K48-linked polyubiquitination; and 63, K63-linked polyubiquitination.

Researches have shown that TRIMs play a critical role in restricting viral replication *via* regulating the innate immune signaling (Masroori et al., 2016; Choudhury et al., 2020). Viruses, however, have developed various strategies to antagonize the antiviral function of TRIM proteins, including improving their proteasomal degradation, affecting their relocation, and ubiquitination (Schilling et al., 2017; Chen et al., 2018a; Koepke et al., 2021). For example, IAV is able to antagonize TRIM25 for its replication (Gack et al., 2009). In this section, we discussed the mechanism of viral evasion by antagonization of TRIMs, including TRIM6, TRIM19, TRIM23, and TRIM25, as depicted in Figure 2.

### TRIM6

TRIM6 restricts viral replication *via* enhancing type I IFN production. It interacts with IKKε and catalyzes the synthesis of unanchored K48-linked polyubiquitin chains, which in turn activates IKKε for subsequent STAT1 phosphorylation, resulting in increasing of IRF3-mediated type I IFN production (Rajsbaum et al., 2014b). This pathway can be evaded by the matrix structural protein (M) of Nipah virus (NiV). The NiV-M protein interacts with TRIM6 and promotes TRIM6 degradation, so the synthesis of unanchored K48-linked polyubiquitin chains and production of type I IFNs are inhibited (Bharaj et al., 2016). It has been reported that the Ebola virus VP35 protein hijacks TRIM6 to promote its ubiquitination and polymerase activity, which results in the reduction of type I IFNs and increase of virus replication (Bharaj et al., 2017).

### TRIM19/PML

TRIM19/PML, also named leukemia (PML) protein, localizes both in the nucleoplasm and in the nuclear bodies (NBs). The function of TRIM19 in viral restriction has been fairly thoroughly investigated, for both DNA viruses and RNA viruses, such as vesicular stomatitis virus (VSV), HSV, IAV, varicella-zoster virus (VZV), HIV, HCMV, and rabies virus (Blondel et al., 2010; Reichelt et al., 2011; Dutrieux et al., 2015; Masroori et al., 2016). The mechanisms of restriction are various and include the silencing of viral genomes, entrapment of newly synthesized nucleocapsids (Ns), indirect interference with reverse transcription, and regulating of type I IFN induction.

On the other hand, some viruses have developed strategies to suppress PML. The HCMV immediate-early protein IE1, for instance, directly binds to the CCD of TRIM19 through its globular core, and it prevents the SUMOylation of TRIM19 and disrupts the nuclear bodies (Scherer et al., 2014). Binding of IE1 to TRIM19 abrogates the *de novo* SUMOylation of PML at specific lysine residues, but this does not affect global protein SUMOylation (Schilling et al., 2017). TRIM19 represses the replication of EV71 by inhibiting autophagy and increasing response to IFNs (Chen et al., 2018a). In EV71-infected Hela cells, the expression of TRIM19 is reduced and the reduction is caused by viral protease 3C^pro^ rather than proteasome pathway (Chen et al., 2018a). The degradation of NB-associated PML has also been identified in HSV-1-infected cells. The HSV-1 immediate-early protein ICP0 directly binds to PML and Sp100, inducing proteasome-dependent degradation (Chelbi-Alix and de The, 1999; Boutell et al., 2003). The ORF75c protein encoded by murine gammaherpesvirus 68 (MHV68) contains ubiquitin E3 ligase activity and mediates direct ubiquitination of PML, resulting in its degradation by the proteasome (Sewatanon and Ling, 2013). Arenaviruses use a different strategy to incapacitate TRIM19: The Z protein of arenaviruses colocalizes with PML *via* the RING domain and induces its relocation from NBs to the cytoplasm (Kentsis et al., 2001) This results in PML and the Z protein binding directly to the translation initiation factor eIF4E and inhibiting translation (Kentsis et al., 2001).

TRIM19 plays an important role against various viral infections, and the restriction of TRIM19 to viruses depends on the antiviral mechanisms induced by IFNs (Nisole et al., 2005; Chen et al., 2018a). Viruses have developed strategies to antagonize TRIM19, but the NB disruption induced by TRIM19 degradation to overcome a cellular antiviral defense remains controversial (Kentsis et al., 2001; Lopez et al., 2002). In PML overexpression cells, ICP0 colocalized with PML in ND10 early in infection, but the two proteins did not overlap or were juxtaposed in orderly structures and PML overexpression had no significant effect on HSV-1 replication (Lopez et al., 2002). It is possible that other virus mechanisms may block PML-mediated repression, and further studies are needed.

### TRIM23

It has been demonstrated that TRIM23 is involved in the induction of autophagy to decrease viral replication (Sparrer et al., 2017). Upon viral infection, the GTP hydrolysis activity of TRIM23 is activated by the autoubiquitination of ARF with unconventional K27-linked polyubiquitin. This facilitates TBK1 dimerization, which proceeds to phosphorylate p62 and ultimately induces autophagic degradation of viral components (Sparrer et al., 2017).

HSV-1 has developed strategy to enhance its replication by antagonizing TRIM23. In HSV-1-infected cells, on the other hand, expression of the HSV-1 Us11 protein promotes HSV-1 growth, while expression of TRIM23 restricts HSV-1 replication in the absence of US11 (Liu et al., 2019). The Us11 protein binds the ARF domain in TRIM23 and disrupts the TRIM23-TBK1 complex, causing decrease of autophagy-mediated restriction of HSV-1 infection (Liu et al., 2019).

### TRIM25

TRIM25 restricts the replication of viruses, such as MERS-CoV, NDV, IAV, SinV, SARS-CoV-2, porcine reproductive and respiratory syndrome virus (PRRSV), and Sendai virus (SeV; Zeng et al., 2010; Sanchez et al., 2016; Hu et al., 2017; Zhao et al., 2019; Choudhury et al., 2020). TRIM25 catalyzes K63-linked polyubiquitination on the N-terminal CARD at K172 of RIG-I *via* its B30.2 domain chains, and this facilitates its recruitment to MAVS and thus induces downstream IFNs induction (Sanchez et al., 2016). However, K48-linked ubiquitination of TRIM25 negatively regulates RIG-I activation, though this can be reversed by the deubiquitinating enzyme USP15 (Pauli et al., 2014). Recently, it was found that TRIM25 enhances the antiviral activity of zinc finger antiviral protein (ZAP) with both ubiquitin ligase activity and multimerization (Li et al., 2017; Zheng et al., 2017). In addition, TRIM25 ubiquitinates DDX3X (a critical component of TBK1) at K55, and TRIM25 and DDX3X cooperatively enhance type I IFN induction following RIG-I activation (Soulat et al., 2008; Atkinson et al., 2021).

TRIM25 plays an important role in a broad range of antiviral activity; however, viruses have evolved a diverse collection of strategies to antagonize TRIM25 activity. The nonstructural protein 1 (NS1) of IAV, for instance, targets the TRIM25 coiled-coil domain directly, thus inhibiting TRIM25 dimerization and preventing the activation of RIG-I with K63-polyubiquitin (Gack et al., 2009). The interaction between NS1 and TRIM25 has been shown to be species-specific, in that human TRIM25 binds to all species-adapted IAV strains, while chicken TRIM25 interacts with NS1 only from avian strains, and murine TRIM25 does not bind with any NS1 interacts (Rajsbaum et al., 2012). However, a recent study of the crystal structures of TRIM25-NS1 complexes showed that the formation of unanchored K63-linked polyubiquitin chains is unchanged by NS1 binding. And the binding of NS1 interferes with the correct positioning of the PRYSPRY domain of TRIM25, which is required for substrate ubiquitination (Koliopoulos et al., 2018). Moreover, the N-terminal domain of NS1 encoded by IBV is responsible for interaction with TRIM25, and this interaction blocks the Lys63-linked ubiquitination of RIG-I (Jiang et al., 2016). Recently, it was found that NS1 disrupts the TRIM25:DDX3X interaction, abrogating both TRIM25-mediated ubiquitination of DDX3X and cooperative activation of the IFNB1 promoter (Atkinson et al., 2021).

Like IAV/IBV, the coronaviruses, including SARS-CoV, MERS-CoV, and SARS-CoV-2, are able to suppress TRIM25 activity *via* the viral N protein. The N protein of SARS-CoV has been demonstrated to interact with the C-terminal SPRY domain of TRIM25, interfering with subsequent ubiquitination of the RIG-I CARD domains and negative regulating type I IFNs (Hu et al., 2017). This strategy of RIG-I-induced IFN-β reduction is also seen in SARS-CoV-2 and MERS-CoV (Hu et al., 2017; Oh and Shin, 2021). In addition to modulating type I IFNs, the N protein of MERS-CoV also suppresses type III expression, and this offers a plausible mechanism for coronavirus evasion of the host immune response (Chang et al., 2020). Interestingly, although not a coronavirus, PRRSV exhibits a similar loss of RIG-I-induced IFN-β when its N protein associates with TRIM25 (Zhao et al., 2019). In foot-and-mouth disease virus (FMDV), it is 3B protein that suppresses type I IFN production and host antiviral response by blocking the interaction between RIG-I and TRIM25, which prevents the TRIM25-mediated, K63-linked ubiquitination of RIG-I (Zhang et al., 2020b).

Severe fever with thrombocytopenia syndrome virus (SFTSV), a highly pathogenic member of the *Bunyavirales*, has developed a different mechanism to inhibit TRIM25. The nonstructural protein NSs of SFTSV interacts with and redistributes RIG-I, TRIM25, and TBK1 into NSs-induced cytoplasmic structures, which circumvents IFN responses (Santiago et al., 2014). Another report demonstrated that NSs specifically trap TRIM25 into viral inclusion bodies and inhibits TRIM25-mediated RIG-ILys-63-linked ubiquitination/activation, contributing to suppression of RLR-mediated antiviral signaling at its initial stage (Min et al., 2020).

The DENV and human papillomavirus Type 16 (HPV16) suppress TRIM25 activity by USP15 to attenuate RIG-I signaling. PR-2B, an epidemic strain of dengue virus, encodes subgenomic flavivirus RNA, which binds to host TRIM25 and prevents USP15-mediated deubiquitination (Manokaran et al., 2015). The E6 oncoprotein of HPV16 interacts with TRIM25 and USP15, promoting K48-linked ubiquitination of TRIM25 and suppressing TRIM25-mediated K63-linked ubiquitination of RIG-I to evade the host antiviral response (Chiang et al., 2018a).

The small t antigen (tAg) of JC polyomavirus (JCV) interacts with TRIM25, preventing it from binding with RNA and inhibiting the K63-linked ubiquitination of RIG-I (Chiang et al., 2021). This antagonism strategy is also conserved in tAg presented polyomavirus BK virus (BKV; Chiang et al., 2021). Herpesviruses use a different strategy to antagonize TRIM25 activity. BPLF1 of herpesvirus promotes the dimerization and autoubiquitination of TRIM25, which inactivates the RIG-I signalosome (Gupta et al., 2018).

## Viral Utilization of Trims

**Figure 3:**
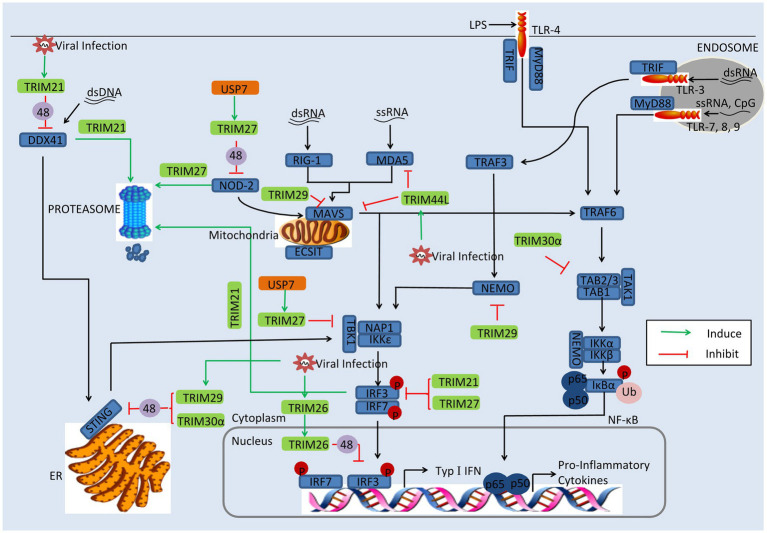
Viral evasion of immune response *via* induction of TRIMs. Some TRIMs (green) negatively regulate innate immune response. Viral proteins (orange) can utilize these TRIMs to reduce the production of type I IFNs and inflammatory factors. USP7, the ubiquitin-specific peptidase 7 of SeV; 48, K48-linked polyubiquitination; and 63, K63-linked polyubiquitination.

Some of TRIMs exhibit negatively regulatory effect in the innate immune. Thus, apart from antagonizing TRIMs, some viruses directly utilize TRIMs or induce their gene expression to promote viral replication. In this section, we discuss viruses employ TRIMs to enhance their replication, including TRIM21, TRIM26, TRIM27, TRIM29, and TRIM30a, as depicted in Figure 3.

### TRIM21

TRIM21, also named Ro52, seems to follow two strategies; some studies indicate that TRIM21 restricts viral replication and positively regulates antiviral pathways, while other reports show that TRIM21 negatively regulates innate immune response to promote viral production (Higgs et al., 2008; Yang et al., 2009; McEwan et al., 2013; Zhang et al., 2013; Manocha et al., 2014). On the one hand, TRIM21 has exhibited antiviral activity. TRIM21 catalyzes the formation of Lys63 (K63)-linked ubiquitin chains and activates the NF-κB, AP-1, and IRF signaling pathways (McEwan et al., 2013). However, in porcine epidemic diarrhea virus (PEDV) infection, TRIM21 exhibits a different strategy; TRIM21 was found to interact and colocalize with the N protein, inducing the degradation of the N protein in a proteasome-dependent manner (Wang et al., 2021).

On the other hand, TRIM21 is also able to facilitate viral evasion of the innate immune response. TRIM21 interacts with IRF3 *via* its C-terminal SPRY domain post-pathogen recognition, resulting in the polyubiquitination and proteasomal degradation of IRF3 (Higgs et al., 2008). The SPRY-PRY domain of TRIM21 targets the DEADc domain of DDX41 at Lys9 and Lys115, inducing the Lys48 (K48)-linked ubiquitination and degradation of DDX41 and thereby inhibiting the innate immune response to intracellular dsDNA (Zhang et al., 2013). Evidence shows that Japanese encephalitis virus (JEV) evades the innate immune response by inducing transcriptional expression of TRIM21 (Manocha et al., 2014). In JEV-infected human microglial cells, TRIM21 overexpression inhibited phosphorylation of IRF3 and activation of IFN-β, while TRIM21 silencing contributed to the type I IFN responses (Manocha et al., 2014).

Other mechanisms involved in antiviral evasion have been reported recently. HPV E7 was found to recruit TRIM21 to ubiquitinate and degrade the IFI16 inflammasome, leading to the inhibition of cell pyroptosis and production of inflammatory factors including IL-18 and IL-1β (Song et al., 2020). SFTSV NSs binds to the carboxylterminal SPRY subdomain of TRIM21, promoting p62 stability and oligomerization and causing activation of the Nrf2 antioxidant signal pathway (Choi et al., 2020). The activation of the p62-Keap1-Nrf2 antioxidant response provides an optimal environment for SFTSV replication (Choi et al., 2020).

### TRIM23

At least two studies have revealed that TRIM23 is an important factor in virus replication (Laurent-Rolle et al., 2014). TRIM23 interacts with and polyubiquitinates yellow fever virus (YFV) NS5 to promote its binding to STAT2 and trigger IFN-I signaling inhibition, thus overcoming the antiviral action of IFN-I (Laurent-Rolle et al., 2014). MnTrim23 identified from *Macrobrachium nipponense* negatively regulates the Relish transcription factor-mediated expression of antimicrobial peptides (AMPs), promoting WSSV replication (Zhang et al., 2020a). It has been also shown that knockdown of MnTrim23 inhibits WSSV replication and VP28 expression.

### TRIM26

Identified as a negative regulator of IFN-β, TRIM26 binds to IRF3 and promotes its K48-linked polyubiquitination and degradation in the nucleus. More important, viral infection promotes TRIM26 nuclear translocation, which in turn increases IRF3 degradation (Wang et al., 2015a). The suppression of IFN-β-involved IRF3 has also been observed in HSV-2, VSV, and PRRSV infection (Huang et al., 2020). It is worth noting that a different report showed that TRIM26 positively regulates type I IFNs, upon RNA viral infection; TRIM26 undergoes autoubiquitination and subsequently associates with NEMO, to promote TBK1–NEMO interaction and the activation of IRF3 and NF-kB (Ran et al., 2016). Some of the discrepancies among these results might be attributed to the different experimental systems (Ran et al., 2016).

### TRIM27

Report mentioned that TRIM27 positive regulates RIG-I signaling, leading to increased production of IFN-β in response to viral infection (Blaine et al., 2015; Conwell et al., 2015). However, other studies have proven that TRIM27 is a negative modulator of immune response. It has been suggested that TRIM27 negatively regulates NOD2-induced NF-κB signaling *via* the K48-linked ubiquitination and subsequent proteasomal degradation of NOD2, which can recognize viral RNA and DNA (Moreira and Zamboni, 2012; Kapoor et al., 2014). In SeV infection there seems to be another mechanism at work; the ubiquitin-specific peptidase 7 (USP7) interacts with and stabilizes TRIM27, promoting the degradation of TBK1 and suppressing the activation of IFN-β (Cai et al., 2018). As in SeV infection, TRIM27 inhibits type I IFN response to HCV infection by inhibiting the IRF3 and NF-κB pathways (Zheng et al., 2019a).

### TRIM29

TRIM29 acts as a negative regulator of proinflammatory cytokines in macrophages and directly binds NEMO, inducing its ubiquitination and proteolytic degradation, which in turn inhibits the NF-κB pathway (Xing et al., 2016). TRIM29 expression is especially induced by DNA virus and cytosolic DNA in macrophages and dendritic cells and targets STING for K48 ubiquitination and degradation, negatively regulating immune response in infection by DNA viruses like HSV-1 and Epstein-Barr virus (Xing et al., 2017; Li et al., 2018). Recently, a different regulatory function of TRIM29 was reported in the context of RNA virus infection in human mDCs; TRIM29 was found to interact with MAVS and subsequently induce K11-linked ubiquitination and degradation, causing decrease of type I IFN (Xing et al., 2018). In addition, TRIM29 ubiquitinates and degrades TAB2, suppressing IFN-γ production in NK cells (Dou et al., 2019). Indeed, deficiency of TRIM29 resulted in an enhanced IFN-γ production and consequently protected mice from murine CMV infection (Dou et al., 2019).

### TRIM30α

TRIM30α enhances the degradation of STING *via* K48-linked ubiquitination at Lys275 in a proteasome-dependent pathway and suppresses innate immune response to DNA viruses (Wang et al., 2015b). TRIM30α also negatively regulates TRAF6-induced NF-κB activation involved in TLR by degrading TAB2 and TAB3 (Shi et al., 2008). Although TRIM30a is well known in mouse, it is phylogenetically related to human TRIMs 5, 6, 22, and 34, which provide the possibility that TRIMs serve as immune regulator in mouse in a similar manner with that in humans (Song et al., 2005).

## Conclusion and Future Perspectives

Here we have collected recent studies on the regulatory effects of TRIMs in several main signaling pathways, and we have discussed the latest research on immune evasion of viruses by antagonizing TRIM activity or utilizing TRIMs. There seems to be no direct correlation between the immune regulation and any particular subfamily of TRIMs. Thus, TRIM6 and TRIM25, belonging to the C-IV group, positive regulate PRR signaling pathways, while TRIM21 and TRIM26, also members of the C-IV group, act as negative regulators of antiviral immune response (Manocha et al., 2014; Rajsbaum et al., 2014b; Ran et al., 2016; Sanchez et al., 2016).

Over the past decade, multiple studies have explored the function of TRIMs in the innate immunity. It is clear that TRIMs play critical roles in regulating immune response to restrict viral infection, especially in NF-κB and IRF signaling. However, TRIMs whose mechanisms are not limited to innate immune regulation. Recently, previously uncharacterized phenomenon has been detected, for example, TRIM25 presentation of RNA-binding activity, TRIM23 induction of autophagy, TRIM21 participation in cell pyroptosis, and TRIM21 activation of the Nrf2 antioxidant signal pathway (Choudhury et al., 2017; Sparrer et al., 2017; Choi et al., 2020; Song et al., 2020). The functions of TRIMs in the innate immunity remain to be further defined, but the functions of TRIMs likely go far beyond immune regulation.

The viral evasions are mainly involved in the regulatory function of TRIMs in immune response. TRIMs, including TRIM25, TRIM6, TRIM14, and TRIM30α (Wang et al., 2015b; Sanchez et al., 2016), act as positive or negative modulator in the innate immune. Nevertheless, reports have shown that some TRIMs are able to regulate immune pathways both positively and negatively, such as TRIM21, TRIM27, and TRIM32 (Higgs et al., 2008; Yang et al., 2009; Conwell et al., 2015). A comprehensive understanding of the regulation of TRIMs on immune response can support further research on antiviral innate immunity pathway dependencies during the process of viral infection. Of highly topical interest, the SARS-CoV, MERS-CoV, and SARS-CoV-2, belonging to the *Coronaviridae* family, have caused devastating pandemic disease in humans. There is evidence to show that the coronaviral nucleocapsid (N) protein suppresses TRIM25 activity to defend against human immune response (Hu et al., 2017; Chang et al., 2020; Oh and Shin, 2021). On the other hand, the N protein can be degraded by TRIM21 (Wang et al., 2021). It is therefore of urgent interest to clarify the dynamic interaction of the N protein with TRIMs, which can provide new clues to understand coronavirus pathogenesis.

The current understanding of the role of TRIMs in immune regulation is broad and well supported, but the knowledge of the mechanisms by which TRIMs participate in viral immune evasion is weakness. This review would be a fruitful line for future investigations. Demonstration of virus-TRIM interactions may reveal new molecular targets for the treatment, or even prevention, of viral infectious diseases.

## Author Contributions

X-ZZ wrote the manuscript. F-HL and X-JW improved the manuscript. All authors contributed to the article and approved the submitted version.

## Funding

This work was supported by the National Natural Science Foundation of China (31772739 and 32172821) and a CAU-Grant for the Prevention and Control of Immunosuppressive Disease in Animals of the China Agricultural University.

## Conflict of Interest

The authors declare that the research was conducted in the absence of any commercial or financial relationships that could be construed as a potential conflict of interest.

## Publisher’s Note

All claims expressed in this article are solely those of the authors and do not necessarily represent those of their affiliated organizations, or those of the publisher, the editors and the reviewers. Any product that may be evaluated in this article, or claim that may be made by its manufacturer, is not guaranteed or endorsed by the publisher.
